# Surviving through a dry spell: microbial responses to drying and rewetting

**DOI:** 10.1128/msystems.00175-26

**Published:** 2026-04-30

**Authors:** Don A. Cowan

**Affiliations:** 1 Centre for Microbial Ecology and Genomics, Department of Biochemistry, Genetics and Microbiology, University of Pretoria 56410, Pretoria, South Africa; CNRS Delegation Alpes, Villeurbanne, Rhône-Alpes, France

**Keywords:** desiccation, anhydrobiosis, transcriptomics, metabolomics

## Abstract

The issues of how microorganisms survive very long periods of desiccation and how they react during both drying and rehydration phases have long been topics of interest in a range of relevant fields, including desert ecosystem microbiomics, food storage, ancient microbe studies, and even astrobiology. The recently published study by Carini et al., who used a combination of transcriptomics and metabolomics to investigate steady-state gene expression and cellular metabolite profiles at different states of bacterial cellular desiccation, during both drying and rewetting phases, adds some valuable insights into how members of bacterial communities can survive in the driest habitats on earth (P. Carini, A. Gomez-Buckley, C. R. Guerrero, M. R. Kridler, et al., mSystems 11:e00493-25, 2026, https://doi.org/10.1128/msystems.00493-25).

## COMMENTARY

The issues of how microorganisms survive very long periods of desiccation and how they react during both drying and rehydration phases have long been topics of interest in the relevant fields, which include desert ecosystem microbiomics (1), food storage (2), ancient microbes (3), and even astrobiology (4).

The recently published study by Carini et al. adds some valuable insights into the functional behavior of bacterial cells during desiccation and rewetting (5). The authors used a combination of transcriptomics and metabolomics to investigate steady-state gene expression and cellular metabolite profiles at different states of cellular desiccation during both drying and rewetting phases. The different states of desiccation were achieved using a simple, elegant (and possibly poorly known) method based on the fact that, at standard temperature and pressure, saturated solutions of various soluble salts generate specific and very different atmospheric humidity levels (6). These humidity levels are typically expressed as water activity (*A*_*w*_) or % relative humidity (%RH) (e.g., KCl, 90% RH; MgCl_2_, 35% RH). Carini et al. used this method to investigate cellular metabolic processes during the drying and subsequent rewetting of *Arthrobacter* cell biomass by successively exposing the biomass samples in a temperature-controlled incubator to different saturated salt solutions. With each change, they allowed sufficient time for re-equilibration at the new atmospheric %RH before samples were recovered for analysis. In the desiccation phase, atmospheric %RH was transitioned from 100% to 25% over a period of 14 days (48 h at each new %RH), after which cells were exposed to a saturated atmosphere for a further 48 h of rehydration.

A limited number of similar studies using mRNA expression profiling to observe changes in metabolic processes during the drying and rewetting phases have been published, but many of these have been performed on eukaryotes, including invertebrates (e.g., see reference 7) and resurrection plants (e.g., see reference 8). Relatively few have focused on bacterial taxa (see table in reference 9; the most recent studies are summarized in Table 1). It is notable that most of these studies (e.g., see references 10–15) have generally only applied two states: saturated and “desiccated,” giving Carini et al., with their sequential desiccation strategy, a rather unique element and adding valuable dimensions to how microbial metabolic processes respond to both progressive drying and subsequent wetting.

**TABLE 1:** Recent studies on gene expression in desiccated microorganisms

Organism	Methodological approach	Key findings	Reference
*Salmonella enterica*	Cells incubated at 100% and 11% RH, gene expression monitored by RNA-seq	Virulence genes *sopD* and *sseD* were linked to cellular desiccation tolerance.	(13)
*Pseudomonas putida*	A comparison of native, desiccated, and rewetted cells; gene expression monitored by RT-qPCR	Cells showed continued gene expression in a desiccation-induced viable but not culturable state.	(14)
*Chronobacter sakazakii*	A comparison of saturated and partially desiccated cells; gene expression monitored by RNA-seq and RT-qPCR	Trehalose biosynthesis pathway genes were highly upregulated during desiccation	(15)
*Pseudomonas putida*	Cells incubated at 50% RH and rewetted; gene expression monitored by microarray and RT-qPCR	Resuscitation from a desiccation-induced VBNC state involved activation of phenylalanine/tyrosine catabolism.	(16)

Responses to drying showed reasonably typical metabolic changes, many of which were related to compatible solute biosynthesis (and acquisition) and stress response processes (ROS protection and DNA repair, in particular). Similarly, observed expression changes during rehydration were broadly consistent with previous studies. The most dramatic changes were broadly associated with the expected reactivation of protein synthesis. Multiple transcripts were associated with the mitigation of oxidative stress, the reactivation of DNA repair processes, and the generation of the C and N building blocks needed for protein and nucleic acid synthesis. The presence of substantial numbers of non-annotated transcripts in desiccation-linked transcriptome modules offers the intriguing potential of novel mechanisms of desiccation tolerance.

Although the authors identified very substantial temporal changes in expression profiles, their analyses do not specifically identify sequential changes in metabolic capacity during the desiccation phase, i.e., evidence for the effective “shutdown” of specific metabolic processes at defined levels of water activity. There is scant evidence in the literature for such processes (summarized in reference 1), although it is generally accepted that higher-order biological functions, such as cell division and photosynthesis, are inactive at water activities below approx. 0.7 or even higher (17).

An interesting ecological aspect of this study, addressed in some detail by the authors, comes directly from the experimental design; i.e., cells were effectively rehydrated by water vapor. The issue of water acquisition in microbial populations in hyperarid desert soils, where liquid water input (principally rainfall) may only occur intermittently, on decadal timescales, has been a matter of some debate (18). One of the general assumptions has been that desert soil microbiomes, which are typically embedded in EPS biofilm matrices, acquire water from the soil atmosphere via the intervention of hygroscopic EPS. While this is certainly a viable process (19), the capacity of cells to also adsorb water directly from the vapor phase is of considerable ecological importance.

The general consensus (1) is that under severely desiccating conditions, microorganisms either form highly resistant resting bodies (spores, akinetes, etc. [20]) or, for non-sporulating taxa, enter a state of dormancy (21). Recent studies have suggested that these states may not fully represent natural desiccating systems. A metatranscriptomics analysis of microbiomes in hyperarid desert soils (22) suggested that while the majority of taxa were effectively inactive (dormant) under these conditions, a small proportion of extant taxa showed significant gene expression levels associated with core processes of N assimilation and heterotrophic C acquisition. The study of Carini et al. does, however, raise questions on the validity of this conclusion. Nevertheless, other, more fundamental questions also remain unresolved. For survival over very long periods, it is assumed that cells require a source of “maintenance energy” to support basic survival processes, such as DNA repair (e.g., see references 23 and 24). The conceptual discrepancy between a state of complete metabolic inactivity (dormancy) and the ongoing need for basal metabolic processes remains something of an anomaly. Use of the rather historic term ”anhydrobiosis” (25) to describe this state does little to clarify the underlying mechanisms.

The study by Carini et al. sheds little light on this intriguing issue. In the fully desiccated state, both the mRNA and metabolite profiles of *Arthrobacter* were shown to be essentially “frozen.” The absence of indicators of metabolic activity in a state of cellular desiccation suggests that this particular organism belongs to the majority of taxa in desiccated habitats: those that effectively shut down cellular metabolism when fully desiccated, rather than retaining a background degree of basal metabolism.

The authors do, however, raise an important issue relating to the interpretation of metatranscriptomic data derived from biomass in desiccated environments. Their observation that fully desiccated, presumably dormant cells retain significant amounts of extractable RNA challenges the paradigm that mRNA can be used as a reliable proxy for microbial activity. Their view, supported by their data, is that the RNA content of desiccated cells represents “the transcriptional state when cells entered dormancy, rather than ongoing transcription.”

The authors discuss the limitations of this study in some detail. In particular, they highlight the assumptions inherent in this experimental system. Cellular cytoplasm is assumed to be at water activity equilibrium with the external atmosphere, but the intracellular water content was not (nor can easily be) accurately quantitated. They also make a valid point that this is just one organism, with a well-known desiccation tolerance. Similar studies with a range of other taxa, with different degrees of desiccation tolerance, are needed to establish whether this study has identified taxon-specific or generic cellular response patterns.

There is, however, a nagging uncertainty: the authors interpret the stability of the transcript and metabolic composition of the *Arthrobacter* cells as indicative of a state of complete dormancy. However, it does seem possible that a residual level of cellular transcription, coupled with an equivalent degree of RNA turnover, might not be distinguishable against a background of stable RNAs. Put another way, cells might possibly retain some functionality under steady-state conditions. It would be particularly interesting to undertake a closer inspection of the “stable RNA fraction,” to interrogate the possibility that a fraction of these transcripts might be linked to processes involved in the generation of sufficient energy for maintenance of long-term cellular survival (23).
